# Storage Conditions of Conjugated Reagents Can Impact Results of Immunogenicity Assays

**DOI:** 10.1155/2016/1485615

**Published:** 2016-07-10

**Authors:** Robert J. Kubiak, Nancy Lee, Yuan Zhu, William R. Franch, Sophia V. Levitskaya, Surekha R. Krishnan, Varghese Abraham, Peter F. Akufongwe, Christopher J. Larkin, Wendy I. White

**Affiliations:** ^1^Drug Metabolism, Pharmacokinetics and Biological Safety Assessment, MedImmune LLC, One MedImmune Way, Gaithersburg, MD 20878, USA; ^2^Analytical Biotechnology Sciences and Strategy, MedImmune LLC, One MedImmune Way, Gaithersburg, MD 20878, USA

## Abstract

Consistent performance of anti-drug antibody (ADA) assays through all stages of clinical development is critical for the assessment of immunogenicity and interpretation of PK, PD, safety, and efficacy. The electrochemiluminescent assays commonly employed for ADA measurement use drug conjugated with ruthenium and biotin to bind ADA in samples. Here we report an association between high nonspecific ADA responses in certain drug-naïve individuals and the storage buffer of the conjugated reagents used in a monoclonal antibody ADA assay. Ruthenylated reagents stored in phosphate-buffered saline (PBS) buffer had increased levels of aggregate and produced variable and high baseline responses in some subjects. Reagents stored in a histidine-sucrose buffer (HSB) had lower aggregate levels and produced low sample responses. In contrast to PBS, conjugated reagents formulated in HSB remained low in aggregate content and in sample response variability after 5 freeze/thaw cycles. A reagent monitoring control (RMC) serum was prepared for the real-time evaluation of conjugated reagent quality. Using appropriate buffers for storage of conjugated reagents together with RMCs capable of monitoring of reagent aggregation status can help ensure consistent, long-term performance of ADA methods.

## 1. Introduction

Protein-based therapeutic drugs have an inherent potential to elicit undesired immune response in human subjects. The impact of treatment-induced anti-drug antibody (ADA) responses may range from inconsequential to potentially life-threatening. Regulatory agencies mandate testing for the presence of ADA in all phases of clinical development and require assessments of potential impact on safety, drug exposure, and efficacy [1–5]. It is therefore crucial to ensure that ADA testing results are accurate and consistent throughout the drug development cycle by implementing long-term maintenance and monitoring of the functional integrity of critical reagents [6–8].

One of the common assay formats for ADA evaluation is the solution phase bridging electrochemiluminescent (ECL) assay, which typically provides high levels of sensitivity and drug tolerance combined with ability to detect most ADA isotypes. In this format, the ECL signal is generated by ADA simultaneously binding two different conjugated forms of the drug: one biotinylated and one conjugated with a ruthenium complex. Conjugation chemistry requires the protein being labeled to be in a buffer free of primary and secondary amines. To achieve this, proteins are typically buffer-exchanged into phosphate-buffered saline (PBS) prior to the chemical reaction. For convenience, conjugated proteins are frequently maintained in the PBS buffer after labeling since PBS is compatible with a large variety of analytical methods, including those used to determine protein concentration. The use of PBS for long-term storage of proteins is fairly common as evidenced by the many commercially available antibodies formulated in PBS and stored at −20°C or below.

While PBS is convenient and widely used, numerous literature reports demonstrate that this buffer is far from ideal for cryostorage of proteins. Freezing of sodium phosphate buffers leads to precipitation of dibasic sodium salts which in turn causes a significant drop of pH. For example, pH of a 50 mM sodium phosphate solution may drop from 7.00 when measured at 25°C down to 3.36 when measured at −30°C ‎[9]. In addition, formation of the Na_2_HPO_4_·12H_2_O crystals leads to a local increase of protein concentration due to sequestration of water from the solution ‎[10]. Localized high protein concentration combined with low pH and the presence of the liquid-solid interface on the surface of the dibasic sodium phosphate crystals may stimulate protein unfolding and aggregation [11]. Problems related to precipitation of dibasic sodium phosphate crystals may be eliminated by the use of 50 mM potassium phosphate containing 6.5% sucrose (a cryoprotectant), which was proposed by Staack et al. as an adequate buffer for long-term cryostorage of most antibodies [6]. As an alternative to cryostorage, long-term refrigeration of proteins at 5°C can eliminate problems caused by aggregation; however it presents a separate set of challenges such as potential for microbial contamination, protein hydrolysis reactions, and possibility of assay interference caused by the use of protein stabilizers (e.g., bovine serum albumin).

It should be remembered that even well-developed formulation buffers may not be able to completely eliminate protein degradation and formation of protein aggregates which are driven by a complex interplay between the storage temperature, protein concentration, and formulation buffer components as well as by the rate of cooling/thawing and the storage container material and size [12, 13]. Selection of formulation buffers suitable for long-term storage of critical reagents used in ADA assays may be of crucial importance due to emerging evidence that aggregation of conjugated reagents can play a critical role in generation of reliable immunogenicity data [14].

An ECL solution phase bridging assay on Meso Scale Diagnostics (MSD) platform for detection, confirmation, and titration of anti-drug antibodies against a therapeutic human monoclonal antibody was developed and validated by MedImmune. The method was subsequently transferred to a second laboratory site to support clinical studies for two disease indications (diseases A and B). The initial assay transfer was deemed successful based on the performance of the provided quality control samples: negative controls and low and high level ADA positive controls gave responses within the ranges established during the original method validation when tested at the second site. Unexpectedly, however, screening and confirmatory cut points obtained at the second laboratory site were significantly higher than those obtained during the original method validation in the same disease populations. For both disease A and disease B, the responses of serum samples from drug-naïve subjects were more variable and many were much higher than those observed during original validation. High responses were detected only in certain individuals but not in the negative control pool which suggested that the high sample-to-sample variability could be caused by aggregation of conjugated reagents similar to that reported by Tatarewicz et al. [14]. Tatarewicz and colleagues demonstrated that the presence of very small amounts (0.55%) of high molecular aggregates in the preparations of ruthenylated monoclonal antibody used in a bridging electrochemiluminescent immunogenicity assay caused a significant increase of baseline ADA responses in subjects with rheumatoid arthritis. While both biotinylated and ruthenylated reagents showed a similar level of aggregation, it was the ruthenylated conjugate that was found to be chiefly responsible for elevated signal. Based on this report and our own accumulated experience with the ECL immunogenicity assays, we evaluated reagent aggregation and its impact on ADA assays focusing on the ruthenylated conjugate as being more critical for assay performance than its biotinylated counterpart.

We performed an investigation into the effects of different formulation buffers on aggregation of ruthenylated antibody and the resulting ADA responses of drug-naïve subjects. Two storage buffers were compared: phosphate-buffered saline (PBS: 3 mM dibasic sodium phosphate, 1 mM potassium phosphate, and 155 mM NaCl, pH 7.4) and histidine-sucrose buffer (HSB: 25 mM histidine, 250 mM sucrose, pH 6.0) as examples of formulations that may, respectively, promote and prevent protein aggregation. Our selection of the histidine-sucrose buffer was driven by the relative ease of its preparation and its general similarity to most formulation buffers used for long-term cryostorage of monoclonal antibodies. We found that relatively small amounts of aggregate present in the ruthenylated reagent stored in PBS caused a disproportionately high increase of nonspecific signal in some individual serum samples. Replacing PBS with histidine-sucrose buffer (HSB) effectively eliminated protein aggregation and significantly reduced the amount of sample-to-sample variability in the immunogenicity assay. We also developed a reagent monitoring control comprised of pooled individual sera that are sensitive to the aggregation level of conjugated reagents and implemented it for monitoring of reagent quality during long-term clinical studies.

## 2. Materials and Methods

### 2.1. Reagents

Positive ADA assay controls were generated by MedImmune and consisted of affinity-purified goat polyclonal antibody directed against the idiotype of the human monoclonal antibody drug. Individual human serum samples and pooled human serum were obtained from Bioreclamation (Hicksville, NY). Multiarray high binding streptavidin-coated plates (MA6000), Blocker A, Read Buffer 4XT, and ruthenium (II) sulfo-trisbipyridine N-hydroxysuccinimide ester (Sulfo-Tag) were obtained from Meso Scale Diagnostics (Gaithersburg, MD). EZ-Link biotin sulfo-N-hydroxysuccinimide ester and 40 kDa Zeba spin desalting columns were purchased from Life Technologies. G75 Sephadex was purchased from Sigma-Aldrich. All antibody and antibody conjugates concentrations were measured using Pierce BCA protein assay kit with bovine gamma globulin standards (Life Technologies). Histidine was purchased from JT Baker, and sucrose was purchased from EMD Millipore.

### 2.2. Conjugation of the Therapeutic Antibody

Prior to conjugation, the human therapeutic monoclonal antibody was buffer-exchanged into PBS (3 mM dibasic sodium phosphate, 1 mM potassium phosphate, and 155 mM NaCl, pH 7.4), using a 40 kDa Zeba spin desalting column. Conjugations were performed by treating a 2 mg/mL solution of the antibody for 1 hour at room temperature either with NHS-Sulfo-Tag at a 15 : 1 challenge ratio or with sulfo-NHS-biotin at a 10 : 1 challenge ratio. Ruthenylated antibody was split into two portions; the first portion was purified using a 70 mL bed volume of G75 Sephadex media equilibrated with PBS, pH 7.4. The PBS eluate was diluted to 1 mg/mL with PBS and stored as single use aliquots (250 *μ*L/vial) at −80 ± 10°. The second portion was purified using a 70 mL bed volume of G75 Sephadex media equilibrated with 25 mM histidine·HCl, pH 6.0. The eluate in the 25 mM histidine buffer was diluted to 1 mg/mL using the same buffer and concentrated sucrose solution so that the final buffer formulation was 25 mM histidine and 250 mM sucrose, pH 6.0 (henceforth referred to as histidine-sucrose buffer or HSB). Biotinylated antibody was purified using a 70 mL bed volume of G75 Sephadex media equilibrated with 25 mM histidine·HCl, pH 6.0. The eluate was diluted to 1 mg/mL with HSB and stored as single use aliquots (250 *μ*L/vial) at −80 ± 10°. For normal use, individual aliquots of both ruthenylated and biotinylated reagents were thawed and stored at 5 ± 3°C for up to one month before being discarded.

### 2.3. Stability Study Design

Ruthenylated antibody solutions prepared in PBS buffer and HSB were placed in 500 *μ*L polypropylene cryovials equipped with O-ring screw caps at 250 *μ*L/vial. The aliquots were subjected to up to 5 freeze/thaw cycles. Each cycle consisted of freezing the aliquots for approximately 16 hours (overnight) at −80 ± 10°C (nominal) and then thawing them at 5 ± 3°C (nominal) for approximately 8 hours. Control aliquots (never frozen) were stored at 5 ± 3°C. Control aliquots and aliquots subjected to 1, 3, and 5 freeze/thaw cycles for both buffer formulations were analyzed by High Performance Size-Exclusion Chromatography (HPSEC) and used in the immunogenicity assay.

### 2.4. High Performance Size-Exclusion Chromatography

Levels of aggregation were assessed by HPSEC on an Agilent HPLC-1200 system (Agilent Technologies, Santa Clara, CA) operated by Agilent OpenLab software. High resolution TSKgel Super SW mAB HR column (4 *μ*m, 250 Å, 7.8 mm ID × 300 mm) from Tosoh Bioscience (Tosoh Bioscience, Montgomeryville, PA) was isocratically eluted by 0.1 M sodium sulfate and 0.1 M sodium phosphate buffer, pH 6.8, and protein elution was monitored by UV absorbance at 280 nm. Wyatt Technologies DAWN EOS MALS detector coupled with UV detection was used in Size Exclusion Chromatography Multiangle Light Scattering (SEC-MALS) analyses. Structure designations were made based on comparisons with stressed antibody reference standard analyzed by SEC-MALS. Column performance was verified by running a gel filtration standard protein mixture (Bio-Rad, Hercules, CA).

### 2.5. Immunogenicity Assay and Cut Point Determination

Serum samples were diluted 50-fold with assay buffer (phosphate-buffered saline/10% MSD Blocker A/0.1% Tween 20, pH 7.2) containing a mixture of the ruthenylated and biotinylated forms of the drug at 0.8 *μ*g/mL and 0.4 *μ*g/mL, respectively. Samples were incubated for 17–22 hours with gentle agitation at room temperature (RT) in the dark. High binding streptavidin-coated (HB-SA) 96-well MSD plates were blocked with MSD Blocker A (150 *μ*L/well) for approximately 2 hours at RT and washed four times with wash buffer (PBS/0.1% Tween 20, pH 7.2). The reaction mixture was transferred to the blocked HB-SA plate (50 *μ*L/well) and incubated for 1 hour at RT with gentle agitation in the dark. Plates were washed four times with the wash buffer prior to addition of 2x MSD Read Buffer T (150 *μ*L/well) and read immediately on MSD Sector 6000 Imager. Screening and confirmatory evaluations were performed on the same assay plate, each in duplicate wells. Confirmatory wells were treated the same as the screening assay wells except for the addition of excess unconjugated drug in the overnight incubation step (final concentration 10 *μ*g/mL). Screening and confirmatory data were obtained by testing 48 drug-naïve human serum samples each from disease A and disease B patient populations using conjugated reagents stored in PBS or in HSB each exposed to 1 F/T cycle. Different sets of A and B disease samples were used for each reagent storage buffer. Each sample was tested in duplicate wells in four separate experiments performed by two different operators on two different occasions, generating a total of 192 screening and confirmatory data points for each disease indication. Each assay plate contained a set of system suitability controls consisting of a negative control measured in four duplicate wells (NQC) and low and high ADA positive controls, each measured in two duplicate wells (LQC and HQC: 15 ng/mL and 1000 ng/mL, resp., of goat anti-idiotypic antibody in NQC). The NQC consisted of a commercial serum pool collected from multiple healthy human male and female donors.

The screening assay responses were expressed as S : B (signal to baseline) values by dividing the mean signal of an individual sample by the mean response of NQC measured on the same assay plate. Confirmatory assay percent inhibition values were calculated as % Inhibition = 100%  [1 − (Response  in  the  presence  of  drug)/(Response in the absence of drug)]. The screening and confirmatory cut points were obtained with the respective false positive rates set to 5% and 1% using statistical methods described in detail elsewhere [15, 16].

### 2.6. Preparation of the Reagent Monitoring Control

Individual serum units from patients diagnosed with disease B (160 in total) were analyzed in the screening assay using ruthenylated and biotinylated reagents stored in PBS and subjected to one freeze/thaw cycle. Sera with S : B signals at or above the original validation screening cut point (31 in total) were retested using both conjugated reagents stored in PBS and in HSB. Samples that changed classification from negative when tested using reagents in HSB to positive when tested with reagents stored in PBS and also showed increase of the S : B signal by >30% (10 in total) were pooled together to create a reagent monitoring control (RMC). The RMC was aliquoted into single use vials and stored at −80 ± 10°C.

## 3. Results

### 3.1. Aggregation of Ruthenylated Antibody

**Figure 1:**
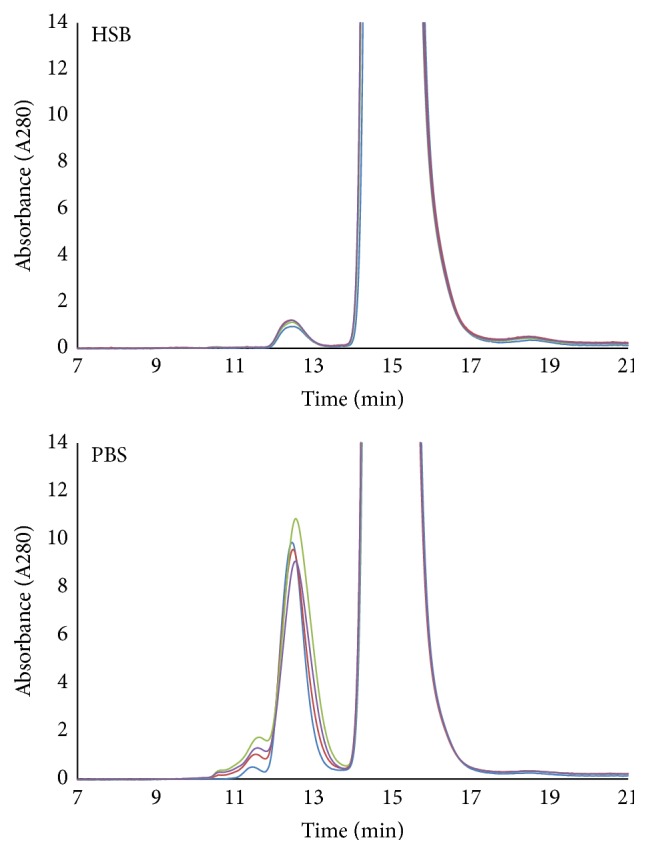
HPSEC traces of ruthenylated antibody conjugate stored at 1 mg/mL in PBS or in HSB. Each preparation was subjected to multiple freeze-thaw cycles by cycling between −80°C (approximately 16 hours) and 5°C (approximately 8 hours) prior to analysis by HPSEC: the light blue color: 0 F/T cycles; the red color: 1 F/T cycle; the green color: 3 F/T cycles; and the purple color: 5 F/T cycles. The retention times, structural designations, and peak areas are shown in Table 1.

Chromatograms and results of the HPSEC analysis of the ruthenylated antibody stored in HSB or in PBS and subjected to 0 (stored at 5 ± 3°C), 1, 3 and 5 freeze/thaw (F/T) cycles are shown in Figure 1 and Table 1.

**Figure 2:**
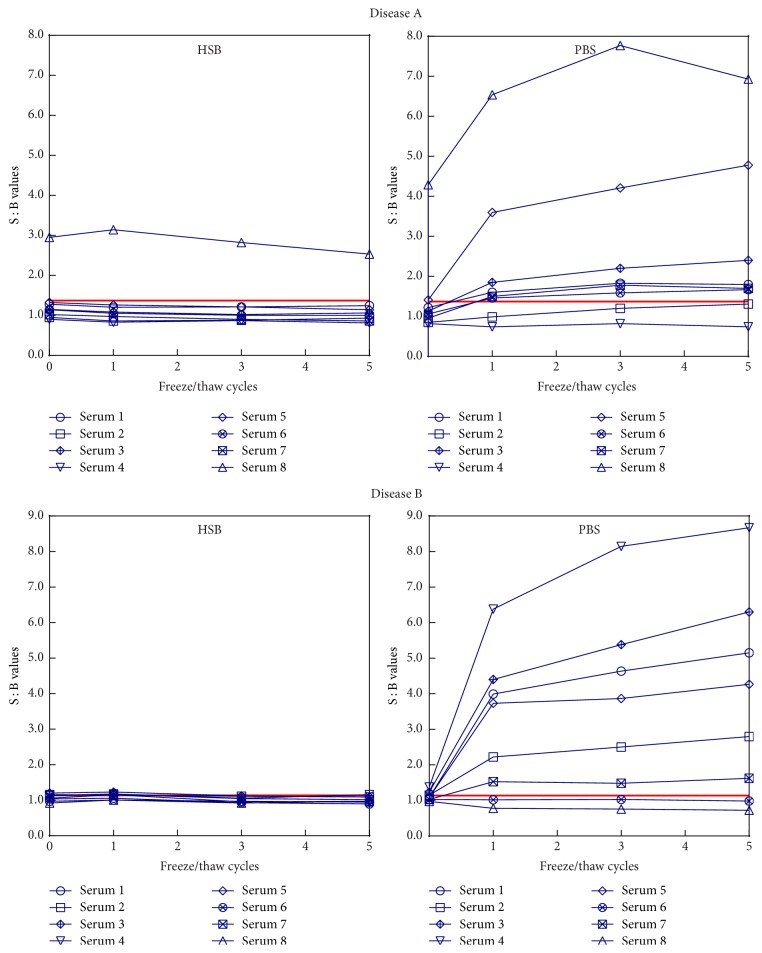
Screening responses of individual disease A and disease B serum samples tested using ruthenylated antibody reagents stored in PBS and in HSB. The solid red line on each graph indicates screening cut point values for disease A (1.37) and disease B (1.14) populations obtained during method validation.

Ruthenylated antibody stored in HSB was 99.6% monomeric and contained about 0.2% of dimers/trimers with no unordered multimers. It was very stable and showed virtually no changes of aggregate and monomer content upon repeated freeze/thaw cycles. On the other hand, the preparation stored in PBS contained unordered multimers (peak at 11.45 minutes with molecular weight of approximately 1900 kDa) and had >10 times more dimers/trimers (peak at 12.45 minutes with molecular weight of approximately 360 kDa) than the HSB preparation, even prior to freezing. The area of the dimers/trimers peak remained relatively unaffected by the number of freeze/thaw cycles and ranged between 2.6% and 3.6% of the total protein. The unordered multimer peak, however, increased with the number of freeze/thaw cycles starting with 0.09% prior to freezing and growing to 0.44% after three freeze/thaw cycles. The rise of unordered multimer content upon repeated freeze/thaw cycles correlated well with the increased responses in the immunogenicity assay (see Figure 2) suggesting that these larger aggregates may be directly responsible for the elevation of ECL signal intensity.

### 3.2. Impact of Reagent Aggregation on the Screening Assay Results

Serum samples from drug-naïve individuals (8 from disease A and 8 from disease B) were tested in the screening assay alongside a set of system suitability controls: NQC, LQC, and HQC. All assays utilized biotinylated antibody reagent prepared in HSB and stored at −80 ± 10°C until time of use. The samples and controls were analyzed using ruthenylated antibody stored in HSB or in PBS that was subjected to 0, 1, 3 and 5 freeze/thaw cycles, and all four freeze/thaw cycles were analyzed on the same plate. The S : B value for each sample was calculated by dividing its mean response by the mean response of NQC analyzed using ruthenylated reagent subjected to the same number of freeze/thaw cycles (Figure 2).

**Figure 3:**
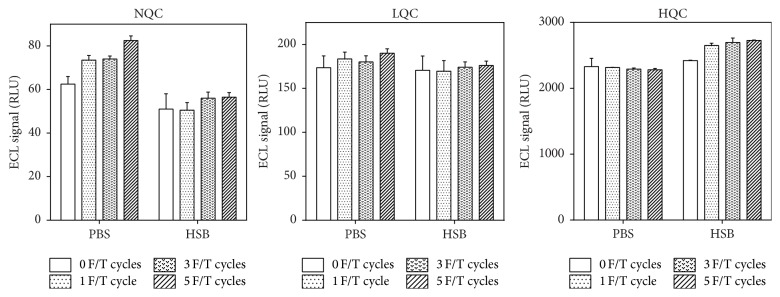
ECL signal in relative light units (RLU) of the system suitability controls: negative control (NQC: pooled serum) and low and high positive controls (LQC: 15 ng/mL, HQC: 1000 ng/mL of affinity-purified anti-idiotype goat polyclonal antibody in NQC) analyzed with reagents stored in PBS and HSB subjected to multiple freeze-thaw (F/T) cycles. Error bars show mean with standard deviation.

The signal to baseline (S : B) responses of samples tested with ruthenylated antibody prepared in HSB were very consistent and were not affected by the number of freeze/thaw cycles applied to the ruthenylated reagent. For all samples in both disease groups the absolute percent difference of response across 5 F/T cycles of ruthenylated reagent was ≤14.7%. Of the eight disease A samples, one was consistently classified as positive and one measured slightly above the screening cut point only with the 0 freeze/thaw cycles' reagent preparation. All eight disease B samples were consistently classified as negative. A dramatically different outcome was observed when the immunogenicity assay was performed with the ruthenylated drug stored in PBS. While only two of eight disease A samples were classified as screen positive for ADA using 0 F/T cycles' reagent, six measured positive after the first F/T cycle. For disease B samples, this number changed from one positive classification at 0 F/T cycles to six after the first F/T cycle. For the majority of samples, the change in classification status was accompanied by a severalfold increase of the S : B signals, most of which occurred upon the initial freezing of the ruthenylated antibody stored in PBS. The increased S : B ratios were due to increased sample responses and not to reduction of the NQC response (see Figure 3). In both disease A and disease B sets there were two samples whose responses were only slightly affected by the reagent storage in PBS. Overall, when the 0 F/T cycles' ruthenylated reagent was used to test the 16 samples, one measured ADA positive using the HSB preparation (6.3%) compared to 12 using the PBS preparation (75.0%). Storage of the ruthenylated reagent in PBS buffer or in HSB did not have an equally dramatic effect on the % inhibition values (data not shown). However, it should be noted that testing of samples with the reagent stored in PBS exposed to 0 and 5 freeze/thaw cycles resulted in changes of the positive/negative classifications of individual samples as well as the overall number of positive classifications. Three disease A samples (numbers 1, 5, and 8) and two disease B samples (numbers 4 and 6) were above the confirmatory cut point (29.9% and 21.2% for diseases A and B, resp.) when tested with the ruthenylated reagent stored in PBS at 0 F/T cycles. After exposure of the ruthenylated reagent to 5 F/T cycles, three disease A samples (numbers 3, 6, and 8) and no disease B samples were confirmed as positive. On the other hand, confirmatory responses for samples tested with the ruthenylated reagent stored in HSB remained constant after 5 freeze/thaw cycles with only two disease A samples (numbers 1 and 8) consistently classified as confirmed positive.

Unlike individual sera, control pool responses were not significantly impacted by the use of different storage buffers for the ruthenylated reagent. Application of multiple freeze/thaw cycles to the ruthenylated reagent did not produce a strong effect on responses of the assay quality controls (Figure 3). After 5 freeze/thaw cycles, the ECL signal of the low and high positive controls remained within ±12.7% of the values obtained with fresh (i.e., never frozen) reagents stored in either PBS buffer or in HSB. A more pronounced effect was observed for the negative control where 5 freeze/thaw cycles applied to the ruthenylated reagent stored in PBS led to a 30% increase of the signal generated with fresh reagents. This increase of the NQC signal is relatively small and cannot account for the observed severalfold upsurge of the S : B responses for some individual samples. It should be noted that, despite a positive trend in signal intensity of the assay controls related to the number of F/T cycles applied to the ruthenylated reagent, all responses remained within acceptable ranges established during the original method validation. Negative and positive control pools did not respond to changes in the conjugated reagent integrity and, therefore, are insufficient to monitor increases in sample responses associated with reagent aggregation.

### 3.3. Reagent Monitoring Control

**Figure 4:**
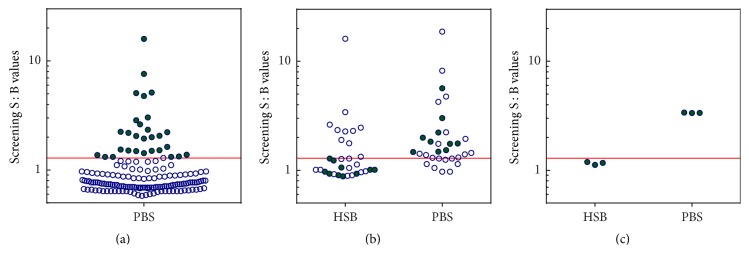
Preparation of the reagent monitoring control. (a) 160 commercial serum samples from drug-naïve subjects diagnosed with disease B were screened using conjugated reagents stored in PBS and subjected to one freeze/thaw cycle. Solid green circles indicate samples with S : B responses at or above the screening cut point. (b) Samples selected in step (a) were tested in the screening assay using conjugated reagents stored in PBS and in HSB. Solid green circles indicate samples that changed classification from negative when tested using reagents in HSB to positive using reagents in PBS. (c) Samples identified in step (b) were pooled together and measured three times using conjugated reagents stored in HSB and using reagents in PBS. Green circles in this panel represent the three separate measurements of the final RMC pool. Red line indicates assay cut point for disease B.

As shown in Figure 3, a regular set of system suitability controls may be inadequate to detect degradation of conjugated reagents and the resulting increase of positive classifications. In order to prepare a control sensitive to aggregation status of the conjugated reagents, we focused on disease B population where the sample-to-sample differences caused by the presence of aggregates were more common and more striking (Figure 4).

We screened 160 individual serum units from individuals with disease B using conjugated reagents in PBS (1 F/T cycle) and selected 31 samples with responses above the screening cut point. These samples were retested following the same procedure and on the same assay plate but using ruthenylated reagents stored in either PBS or HSB in order to identify sera that changed ADA classification status (negative to positive) and showed increase of the S : B signal by >30%. Ten samples met these criteria and were pooled to create a reagent monitoring control (RMC). When tested with aggregated reagents (in PBS), RMC responses increased approximately 3-fold in comparison to the result obtained with nonaggregated reagents (in HSB) thus demonstrating that RMC could be used to monitor reagent aggregation status during clinical studies.

### 3.4. Cut Point Determination in Drug-Naïve Disease A and Disease B Populations

**Figure 5:**
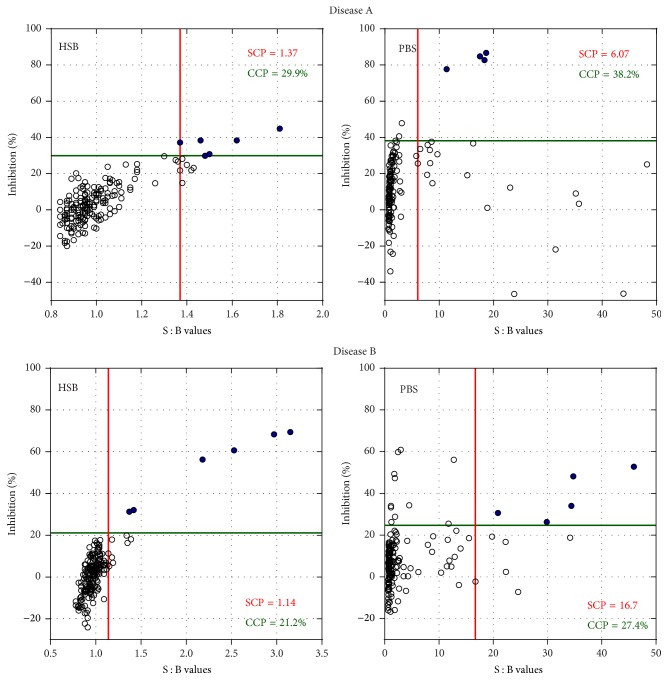
Screening and confirmatory responses of individual drug-naïve disease A and disease B samples tested using conjugated reagents stored in HSB or in PBS. The vertical red and horizontal green lines on each graph show screening (SCP) and confirmatory cut points (CCP), respectively. Open circles (○) indicate negative responses and closed circles (●) correspond to confirmed positives.

The large differences in sample responses observed across different reagent storage buffers led us to suspect that even slight aggregation of the conjugated reagents may have a tremendous impact on the screening and confirmatory cut point values and, consequently, on the reported baseline reactivity in drug-naïve populations. Screening and confirmatory data were generated for disease A and disease B populations using conjugated reagents stored in PBS or HSB, each exposed to 1 F/T cycle. Screening and confirmatory data for the drug-naïve disease A and disease B populations and the resulting cut point values are shown in Figure 5.

As expected, screening cut points established using reagents stored in PBS (and containing aggregates) were much higher than those obtained with reagents stored in HSB (little or no aggregation) with the difference being approximately 4-fold for disease A population and almost 15-fold for disease B population. It should be noted that all cut point values were obtained after removal of statistical outliers following the customary procedures detailed in [15, 16]. The elevated screening cut point values obtained with the PBS reagent preparations were caused by broadening of signal distributions in both disease A and disease B populations. Confirmatory cut points obtained using reagents stored in PBS were also higher than those generated with reagents stored in HSB but the differences were less pronounced than those observed for the screening cut points. Increase of the confirmatory cut point for disease A samples was caused by a combination of broader distribution and increased median, while for disease B population increased median accounted for most of the change in the confirmatory cut point value.

## 4. Discussion

### 4.1. Aggregation of Conjugated Reagents

We conducted a stability evaluation of the ruthenylated monoclonal antibody and in lieu of storing the reagent for several years at <−70°C, which would be expected for its typical use during testing of clinical samples, we chose to subject the reagent to multiple freeze/thaw cycles which may simulate the stress of long-term cryostorage [13, 17]. We evaluated two storage buffers: phosphate-buffered saline (PBS: 3 mM dibasic sodium phosphate, 1 mM potassium phosphate, and 155 mM NaCl, pH 7.4) and histidine-sucrose buffer (HSB: 25 mM histidine, 250 mM sucrose, pH 6.0) in order to, respectively, enhance and reduce protein aggregation. Changes of protein purity caused by storage in these two buffers were monitored by High Performance Size-Exclusion Chromatography. Concentration of aggregates in the preparation of ruthenylated antibody stored in HSB remained constant at about 0.2% through five freeze/thaw cycles which provides assurance that conjugates stored in this buffer should be able to remain in cryostorage for years without any appreciable degradation. PBS proved less efficient in maintaining integrity of the conjugated antibody. An elevated amount of dimers and trimers (2.6%) appeared prior to freezing of the preparation in PBS which implies that certain degree of aggregation may be due to phosphate buffer alone and is not related to protein degradation caused by freezing. This is underscored by the observation that the amount of dimers/trimers was not appreciably affected by the number of freeze/thaw cycles. Species that formed in response to the freeze/thaw induced stress were identified as unordered multimers with molecular weight of about 1900 kDa. Increased concentrations of unordered multimers in the ruthenylated antibody preparation were associated with the increased responses of selected individual samples in both disease A and disease B, suggesting that this species is directly responsible for high ECL responses. Despite formation of aggregates in PBS, it should be noted that the monomer content remained ≥95.9% throughout all five freeze/thaw cycles, which for most antibody applications would be considered acceptable and would not trigger a follow-up investigation. Our observation that the presence of ≤0.44% of high molecular aggregates can have a profound impact on the individual responses in the immunogenicity assay suggests that quality requirements for conjugated reagents used in immunogenicity testing should be more stringent than those typically applied to monoclonal antibodies.

### 4.2. Nature of Matrix Interference

To the best of our knowledge, the publication by Tatarewicz et al. [14] is the only report linking the presence of aggregates in the conjugated reagents used for detection of ADA with high apparent baseline reactivity in a drug-naïve population. This report demonstrates that high signals in the ECL immunogenicity assay could be reduced by modifying the conjugated antibody formulation buffer with sucrose, a cryoprotectant, which was able to prevent formation of aggregates. Their study was performed using samples from patients with rheumatoid arthritis known to contain significant amounts of rheumatoid factor (RF) which may bind to aggregated immunoglobulins; however no correlation between the RF levels and the intensity of response in the ADA assay was detected. We also tested sixteen individual serum samples with RF ranging from 12.2 U/mL to 650.0 U/mL and we did not observe any correlation between the RF level and response in the screening assay using either of the two different formulations of the ruthenylated antibody (data not shown).

Using ruthenylated reagent stored in PBS, we observed high baseline reactivity in drug-naïve samples from patients diagnosed with disease A and disease B, two populations typically not associated with high levels of RF. Similar to the previous report, storage of conjugated reagents in PBS (containing no cryoprotectant) instead of HSB (containing 250 mM sucrose) caused formation of aggregates, which in turn resulted in a dramatic increase of the screening responses in both populations. Inexplicably, this increase was far from uniform: it reached as much as 6-fold for some individual disease A and disease B samples while other samples appeared to be unaffected. It is far from obvious how the aggregates present in the ruthenylated reagent can cause a significant signal increase for certain samples. One could hypothesize that high molecular weight aggregates (composed of 12-13 molecules based on the observed average molecular weight of 1900 kDa) are capable of amplifying ECL signal to a higher degree than a monomeric molecule, thus strongly enhancing otherwise insignificant sample-to-sample differences. Dramatic differences in behavior of individual serum samples in response to aggregated reagents can have a significant impact on screening and confirmatory cut point values as well as the number of positive classifications generated by the immunogenicity assay. Thus, integrity of conjugated reagents becomes of utmost importance for ensuring quality of immunogenicity data as discussed below.

### 4.3. Impact of Reagent Aggregation on ADA Responses in Drug-Naïve Populations

One can envision a scenario where, due to gradual degradation of conjugated reagents, screening and confirmatory responses of some individual samples show a marked increase similar to that illustrated in Figures 2 and 5. Such increases may be misinterpreted as developing immune responses, especially if ADA positive/negative classifications are made using cut points obtained with intact conjugated reagents. For example, screening and confirmatory cut point values generated in disease A and disease B populations using nonaggregated reagents (i.e., stored in HSB) are 1.37 and 29.9% and 1.14 and 21.2%, respectively. As shown in Figure 5, application of these cut points to data generated with aggregated reagents (i.e., stored in PBS) would result in a very high number of confirmed positive classifications. A common practice in clinical studies is to establish in-study cut points using either nontreated commercial samples or pretreatment patient samples prior to immunogenicity testing [18]. However, cut points obtained with aggregated reagents can be very high as shown in Figure 5. Cut point values for disease A and disease B populations obtained with reagents stored in PBS were 6.07 (screening) and 38.2% (confirmatory) and 16.7 (screening) and 27.4% (confirmatory), respectively. Such high screening cut point values would significantly reduce assay sensitivity and drug tolerance and may be invalid for sample testing.

It is expected that loss of reagent integrity should be detected by unacceptable performance of the system suitability controls resulting in assay failure that should preclude reporting of inaccurate immunogenicity results. As shown in Figure 3, however, aggregation of ruthenylated reagents had negligible effect on performance of both positive and negative assay controls, thus raising the likelihood of unwittingly reporting immunogenicity results that are heavily “contaminated” with false positives. In order to eliminate such a possibility, we generated a reagent monitoring control (RMC) by pooling together individual serum samples selected for their ability to strongly increase their ECL signal in response to the presence of aggregates in conjugated reagents. As shown in Figure 4, RMC is classified as negative when nonaggregated reagents (stored in HSB) are used and positive when tested with aggregated reagents (stored in PBS). The observed 3-fold increase of S : B response upon testing with reagents stored in PBS makes RMC more sensitive to reagent aggregation than the traditional set of positive and negative controls and can be used as a real-time functional test of reagent quality to be performed together with testing of unknown samples.

The RMC is included in the confirmatory assay tier (4 replicates tested in the absence of added drug) for the purpose of monitoring the performance of reagents during sample analysis. RMC is expected to be classified as negative and repeated positive classifications of RMC should trigger a halt to sample testing until completion of an investigation into quality of conjugated reagents.

Due to difficulties in its preparation, RMC cannot easily replace the negative control pool but it can be used in addition to regular system suitability controls. RMC or related controls could also be included in functional testing of stress-induced stability of critical reagents and selection of appropriate formulation buffers similar to the approach described by Staack et al. [6]. While we expect that use of optimal formulation buffer and proper storage of conjugated reagents should prevent their degradation and subsequent changes of assay performance, RMC can be used as an additional precaution ensuring high quality of the immunogenicity data.

## 5. Summary

Integrity of conjugated reagents used for measurement of ADA in clinical samples is critical for generation of reliable immunogenicity data. Small amounts of aggregates present in preparations of conjugated reagents may lead to a spurious increase of ADA positive classifications creating an appearance of immune response developing in certain individuals. System suitability controls prepared in normal serum or plasma pools may be unaffected by the presence of aggregates and perform within specification thus failing to alert the investigators about potential problems with the quality of immunogenicity results. Presence of aggregates in conjugated reagents is best detected using individual samples known to be sensitive to reagent aggregation analyzed either individually or being pooled together to create controls for monitoring of reagent quality. Phosphate-buffered saline may be especially ill suited for cryostorage of proteins and its use should be discouraged. Presence of cryoprotectants such as sucrose in formulation buffers may eliminate or at least reduce aggregation of conjugated reagents during long-term storage at <−70°C.

## Figures and Tables

**Table 1:** Summary of ruthenylated antibody analysis by HPSEC. Ruthenylated antibody was stored at 1 mg/mL in PBS) or in HSB. Each preparation was subjected to multiple freeze-thaw (F/T) cycles by cycling between −80 ± 10°C (approximately 16 hours) and 5 ± 3°C (approximately 8 hours) prior to analysis by HPSEC.

Retention time [min]	Molecular weight [kDa]	Structure designation	% peak area							
			0 F/T cycles		1 F/T cycle		3 F/T cycles		5 F/T cycles	
			PBS	HSB	PBS	HSB	PBS	HSB	PBS	HSB
11.45	~1900	Unordered multimer	0.09	0.0	0.22	0.0	0.44	0.0	0.31	0.0
12.45	~360	Dimers, trimers	2.6	0.2	2.9	0.2	3.6	0.2	2.8	0.2
14.89	~150	Monomer	97.3	99.7	96.9	99.7	95.9	99.6	96.9	99.7
